# Early Endosomes Undergo Calcium‐Triggered Exocytosis and Enable Repair of Diffuse and Focal Plasma Membrane Injury

**DOI:** 10.1002/advs.202300245

**Published:** 2023-09-13

**Authors:** Daniel C. Bittel, Jyoti K. Jaiswal

**Affiliations:** ^1^ Center for Genetic Medicine Research Children's National Research Institute 7144 13th Pl NW Washington, DC 20012 USA; ^2^ Department of Genomics and Precision Medicine George Washington University School of Medicine and Health Sciences Washington, DC 20012 USA

**Keywords:** calcium, early endosomes, endocytosis, exocytosis, pore forming toxins, wounds

## Abstract

Cells are routinely exposed to agents that cause plasma membrane (PM) injury. While pore‐forming toxins (PFTs), and chemicals cause nanoscale holes dispersed throughout the PM, mechanical trauma causes focal lesions in the PM. To examine if these distinct injuries share common repair mechanism, membrane trafficking is monitored as the PM repairs from such injuries. During the course of repair, dispersed PM injury by the PFT Streptolysin O activates endocytosis, while focal mechanical injury to the PM inhibits endocytosis. Consequently, acute block of endocytosis prevents repair of diffuse, but not of focal injury. In contrast, a chronic block in endocytosis depletes cells of early endosomes and inhibits repair of focal injury. This study finds that both focal and diffuse PM injury activate Ca^2+^‐triggered exocytosis of early endosomes. The use of markers including endocytosed cargo, Rab5, Rab11, and VAMP3, all reveal injury‐triggered exocytosis of early endosomes. Inhibiting Rab5 prevents injury‐triggered early endosome exocytosis and phenocopies the failed PM repair of cells chronically depleted of early endosomes. These results identify early endosomes as a Ca^2+^‐regulated exocytic compartment, and uncover the requirement of their dual functions – endocytosis and regulated exocytosis, to differentially support PM repair based on the nature of the injury.

## Graphical abstract

Plasma membrane is routinely exposed to diffuse or focal injury, whose repair is facilitated by the cellular compartments. This study identifies early endosomes as a calcium‐regulated exocytic compartment. Regulated exocytosis of early endosome helps repair focal injury, and repair of toxin‐induced diffuse injury to plasma membrane requires both endocytosis, and regulated exocytosis of early endosomes.

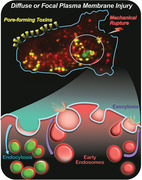

## 1 Introduction

Tissue injury activates spatially and temporally coordinated cellular responses that are tailored to the extent and nature of the injury, failure of which prevents tissue repair.^[^ ^1^ ^]^ Similarly, single‐cell repair requires coordinated subcellular responses that ensure cell survival, and aberrant single‐cell repair results in diseases.^[^ ^2^, ^3^, ^4^ ^]^ Cell injury can be caused by focal trauma that produces a single large localized plasma membrane (PM) lesion, or by chemical agents that produce nanoscale‐sized (diffuse) holes dispersed throughout the PM.^[^ ^4^, ^5^ ^]^ While sub‐nanoscale holes in the PM spontaneously reseal by the redistribution of membrane lipids, repair of nanoscale and microscale injuries require membrane trafficking processes that involve internalization (by endocytosis), shedding (by ectocytosis) or addition of new membrane (by exocytosis).^[^ ^2^, ^5^ ^]^

PM wounding allows extracellular Ca^2+^ influx into the cell that activates the PM repair (PMR) response. Membrane trafficking is central to this response, and is needed to rapidly close the wound and prevent influx of lethal concentrations of extracellular Ca^2+^.^[^ ^5^, ^6^ ^]^ Ca^2+^ binding proteins such as Annexins, Synaptotagmins, Dysferlin, Calpains, and Apoptosis linked Gene‐2 (ALG‐2) that modulate PM dynamics through shedding, bending, constriction, internalization, and exocytic fusion of membranes.^[^ ^7^, ^8^, ^9^, ^10^, ^11^, ^12^ ^]^ Unlike diffuse PM injury which causes Ca^2+^ increase throughout the cell, focal injury causes a polarized Ca^2+^ increase that enables localized activation of repair responses including lysosome fusion, cytoskeleton remodeling, redox signaling, and ion homeostasis by the ER and mitochondria.^[^ ^13^, ^14^, ^15^, ^16^ ^]^ The precise roles of these diverse responses for PM repair, their relative timing, and their mutual interactions remain under active investigation. However, little systematic examination has been carried out to address whether cells utilize common repair mechanisms for all PM injuries or if they mount repair responses tailored to the extent and nature of the PM injury. While endocytosis, ectocytosis, and blebbing are amongst the mechanisms for membrane removal during PMR, Ca^2+^‐triggered lysosome exocytosis is the main mechanism known in mammalian cells for membrane addition to repair focal or diffuse PM injury.^[^ ^3^, ^5^, ^17^, ^18^ ^]^

Yolk granules that fuse with the injured PM of egg cells provide the endomembrane for repairing PM tears, and lysosomes were proposed as the equivalent compartment in mammalian cells that provides endomembrane for mammalian cell PM repair.^[^ ^18^, ^19^, ^20^ ^]^ However, recent studies have identified that injury‐triggered lysosome exocytosis facilitates the repair of focal and diffuse PM injury through exocytic secretion of the lysosomal enzyme Acid Sphingomyelinase (ASM).^[^ ^21^, ^22^, ^23^, ^24^, ^25^ ^]^ Such a role of lysosome exocytosis in PMR is supported by the finding that lack of ASM, or inhibition of its injury‐triggered secretion, both impair PM repair and contribute to degenerative diseases that can be ameliorated by exogenous provision of ASM.^[^ ^9^, ^22^, ^26^ ^]^ ASM secreted by lysosomal exocytosis acts to hydrolyze PM sphingomyelin into a new lipid moiety – ceramide, which induces membrane curvature and facilitates removal of the damaged PM by endocytosis.^[^ ^24^, ^25^ ^]^

The above studies establish that PM turnover by PM endocytosis and endomembrane exocytosis are important membrane trafficking events involved in PMR. Here, we have used time‐lapse imaging of mammalian cells (C2C12 myoblast cell line) undergoing PMR to monitor injury‐triggered membrane dynamics following diffuse and focal injury to the PM. Our analysis identified that acute activation of endocytosis plays a role in PM repair from diffuse, but not focal, PM injury. These efforts led to a serendipitous observation that early endosomes undergo robust Ca^2+^‐triggered exocytosis, and this is required for PM repair, independent of the nature of the PM injury. These findings identify plasma membrane turnover by way of endosome formation, and exocytic fusion, of early endosomes, as two distinct mechanisms through which this cellular compartment facilitates PMR in an injury‐specific manner. We also uncover early endosomes are a Ca^2+^‐triggered exocytic compartment, adding to their known role as the hubs for signaling, cellular communication, and sorting membrane cargoes.^[^ ^27^ ^]^

## 2 Results

### 2.1 Endocytosis is Activated by and Required for Cell Repair from Diffuse PM Injury

**Figure 1:**
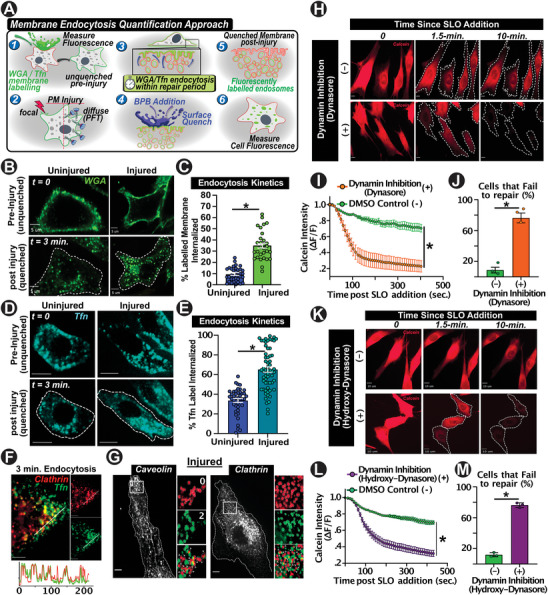
Acute activation of endocytosis is required for repair of diffuse PM injury. A) Schematic depicting bulk membrane endocytosis tracking and quantification with diffuse PFT or focal mechanical injury of the PM using a timed fluorescence PM‐labeling and quenching approach. B,D) Images of B) WGA^AF488^‐labeled cells or D) Tfn^488^‐labeled cells immediately post‐labeling (unquenched) or following quenching of surface label by bromophenol blue (quenched). C,E) Quantification of the proportion of C) WGA^AF488^ or E) Tfn^AF488^ endocytosed in cells that were not injured or PFT‐injured for 3‐min (*p=0.01 C, p = .02 E). F) Image and line intensity plot (bottom) of Clathrin mRFP‐transfected cells following 3‐min of endocytosis of Tfn^AF488^‐labeled PM, demonstrating colocalization and clathrin endocytic uptake route of Tfn label. G) Images of Caveolin‐1 and Clathrin transfected cells prior to PFT injury (inlayed boxes are zoomed in the image on the right with pseudo‐colored panels representing time since injury (red ‐ 0 min and green ‐ 2min). H) Images of Calcein‐labeled cells injured by SLO after treatment either with DMSO (‐) or with dynamin inhibitor Dynasore (+). I) Plot showing kinetics of Calcein dye leak out of cells following SLO‐induced membrane injury in both conditions. J) Quantification of percentage of cells that fail to repair SLO‐injury between conditions. K) Images of Calcein‐labeled cells injured by SLO after treatment either with DMSO (‐) or with 2nd generation dynamin inhibitor hydroxy‐dynasore (+). L) Plot showing kinetics of Calcein dye leak out of cells following SLO‐induced membrane injury. M. Quantification of percentage of cells that fail to repair SLO‐injury (+ Hydroxy Dynasore inhibition). Data in (C,E) represent n = 30‐40 cells per condition (three experimental repeats), data in (I,J,L,M) represent n= 40–50 cells/condition, three replicates per assay. *p < 0.05 versus DMSO control. I, L) Kinetics analyses performed via one‐way repeated‐measures ANOVA with post‐hoc t‐tests and Bonferroni correction. C,E,J,M)Differences in cell repair and endocytosis determined via t‐test for paired comparison. Scale bars (D,F,H,K = 10 µm, B,G = 5 µm). Data indicate mean + SEM.

To monitor PM trafficking during repair of diffuse injury, we used the pore‐forming toxin (PFT) streptolysin‐O (SLO) and titrated its dose to reproducibly injure the PM injury without compromising cell viability (Figure S2A–D, Supporting Information). Treatment of C2C12 cells with 300 U SLO did not induce PM injury (indicated by the lack of FM 1–43 dye influx), while treatment with both 400U and 500 U SLO induced PM injury (Figure S2A–D, Supporting Information). This also identified that 400 U and 500 U of SLO injured >80% of cells independent of the presence of extracellular calcium (Figure S2A–D, Supporting Information). Of the cells that were injured, ≈90% of cells injured by 400U SLO repaired in the presence of extracellular calcium, while only 40% of all the cells injured by 500U SLO were able to repair,  the rest of the cells retracted from the dish and were labeled by the cell impermeant stain propidium iodide (PI) (Figure S2A–D, Supporting Information). These observations identified 400U SLO to be the effective and safe SLO dose for inducing diffuse PM injury. To monitor the dynamics of PM injured by SLO, we labeled the PM using wheat germ agglutinin tagged with alexa fluor (WGA^AF488^) and monitored its trafficking in response to PM injury via time‐lapse confocal microscopy and cell surface fluorescence quenching (**Figure** **1**A).^[^ ^28^ ^]^ This approach enabled reliably quantifying the proportion of PM endocytosed over distinct intervals (i.e., 3‐ and 10‐min), and verify the effect of endocytic blockade by dynamin inhibitors (Dynasore and Hydroxy‐Dynasore) (Figure S1A–C,F, Supporting Information). This revealed that uninjured myoblasts endocytose 10% ± 3% of the PM over a 3‐min period (Figure 1B,C). However, in the 3‐min period post SLO injury, there was over threefold increase in endocytosis (Figure 1B,C). These results were separately confirmed using transferrin as a marker of PM endocytosis, although transferrin internalization rates were noticeably higher for both uninjured and SLO‐injured cells (Figure 1D,E). Subsequently, as we observed increased bulk PM endocytosis (WGA‐labeling) and increased transferrin uptake upon SLO injury, we sought to determine whether SLO injury stimulated particular endocytic pathways. Having confirmed that transferrin was indeed taken up via the dynamin‐dependent clathrin pathway (with its inhibition attenuating transferrin uptake) (Figure 1F; Figures S1I and S3B,A Supporting Information), we discovered that SLO injury activated both Caveolae‐ and Clathrin‐mediated endocytosis (Figure 1G; Figure S2E, S1G,H Supporting Information).

To examine the requirement of endocytosis in PM repair from SLO injury, we inhibited endocytosis by treatment with two forms of dynamin inhibitor – 1st generation Dynasore, and next‐generation Hydroxy‐Dynasore. Using this inhibition approach halted SLO injury‐stimulated caveolin or clathrin endocytosis (Figure S1D,E, Supporting Information). Subsequently, to monitor the kinetics of membrane repair, we labeled cells with calcein dye and tracked dye leakage from the cell upon PM injury, which ceased with successful PM repair. Inhibition of endocytosis rendered the cells incapable of repairing SLO injury to the PM, leading to complete loss of calcein dye, with ≈70–80% of the dynasore‐treated cells failing to repair and detaching from the coverslip (Figure 1H–M). These results identify endocytosis of injured PM is required for PM repair from diffuse SLO injury.

### 2.2 Endocytosis is Acutely Inhibited by Focal PM Injury and is Dispensable for Cell Repair

**Figure 2:**
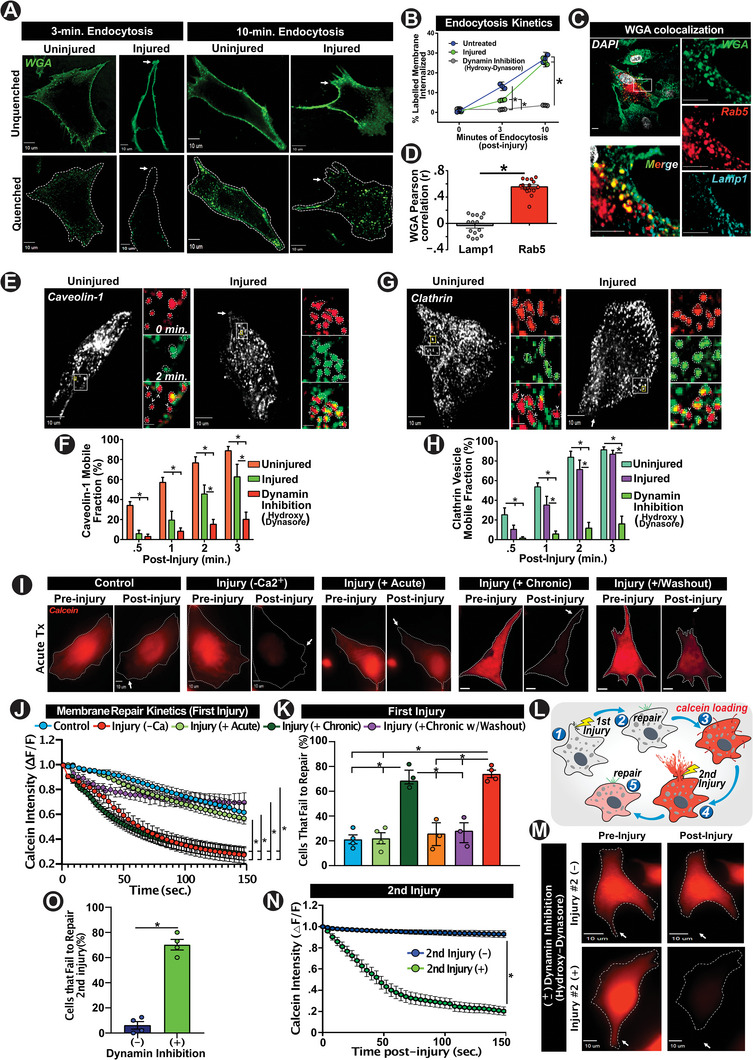
Acute activation of endocytosis is not required for repair of focal PM injury. A) Images showing injured and uninjured cells following surface labeling with WGA^AF^ (upper panel) and quenching of surface WGA^AF^ with bromophenol (lower panel) to monitor bulk endocytosis. B) Plot showing the kinetics of bulk endocytosis in uninjured cells (blue), dynamin‐inhibited cells (gray), and in focally injured cells (green) (*P<0.05 vs. specified condition) (3min. ‐ *Control vs. Injured: p=0.01, Control vs. Dynamin Inhibition: p=.002) (10‐min. ‐ *Control vs. Dynamin Inhibition: p<0.001, Injured vs. Dynamin Inhibition: p<0.001). C) Images and D) Plot showing co‐localization of Rab5‐mCherry (red) expressing cells allowed to endocytose WGA^AF^ (green) for 10‐min and immunostained for lysosomal marker LAMP1 (cyan) (Rab5 vs. LAMP1: *p=0.01). E–H) Images and plots showing kinetics of endocytosis of (E) and (F). Caveolae (Caveolin‐1‐mRFP) and G,H) Clathrin coated vesicle (CCS; Clathrin‐GFP) in cells prior to, or following focal injury. Image show E) Caveolae and G) CCS, with the zoomed images pseudo colored for time – Red, pre‐injury, and Green, 2‐min post‐injury. F,H) Plots quantifying cumulative fraction of Caveolae/CCSs that moved over 3‐min post‐injury respectively. (F) *Control vs. Injured: 0.5‐min; p=0.01, 1‐min.; p=0.01, 2‐min; p=0.02, 3‐min; p=0.02. *Control vs. dynamin inhibition: *p* < 0.001 for all timepoints. *Injured vs. Dynamin Inhibition: 1‐min.; p=0.04, 2‐min.; p=0.02, 3‐min; p=0.01). (H) *Control vs. Injured: 0.5‐min; p=0.01, 1‐min.; p=0.03, 2‐min; p=.04, *Control versus dynamin inhibition: *p* < 0.001 across all timepoints. *Injured vs. dynamin Inhibition: 0.5‐min.; p=0.02, 1‐min.; p=0.01, 2‐min.; *p* < 0.01, 3‐min; p=<0.001. Data represent mean + SEM from 3 independent experimental replicates (n=10 cells per replicate). I) Images of Calcein‐loaded cells pre/post focal injury in cells that are dynamin‐inhibited for <30 min (+ Acute), for 24 h (+ Chronic), or 24 h followed by a 2‐h washout of the inhibitor (+ Washout). J) Plot showing kinetics of Calcein loss following focal injury of cells treated as stated. K) Plot showing fraction of cells that failed to undergo repair – as indicated by complete loss of Calcein dye. Data represents >3 separate experiments (n= 15‐20 cells per condition). L) Schematic for the repeat focal injury experimental design. M) Images showing cells subjected to initial injury followed by Calcein labeling prior to second injury (pre‐injury) or following second injury (post‐injury) in cells treated with (+) or without dynamin inhibitor (‐). N) Plot showing kinetics of Calcein loss following the second focal injury with (+) or without (‐) dynamin‐inhibition. O) Plot showing fraction of cells that failed to undergo repair from second injury leading to total loss of Calcein dye (n= 10‐20 cells/condition, 3‐4 replicates per assay). *P<0.05 vs. denoted conditions. Kinetics analyses in J, N performed via 1‐way repeated‐measures ANOVA with post‐hoc t‐tests and Bonferroni correction. Differences across multiple treatments in in B, F, H, and K were assessed via 1‐way ANOVA. Differences in across pair of treatments in D and N were evaluated via t‐test. Scale bars‐10 µm (insets‐C =5 µm, ‐F, H =1 µm). White arrows= site of injury. Data indicate mean + SEM.

We next monitored PM endocytosis following focal mechanical injury to the PM by laser induced cavitation. Compared to the endocytic rate of 13 ± 3% of total PM over 3 min in uninjured cells, focally injured cells showed reduced endocytosis of 6 ± 2% of total PM during the 3 min following injury, and mere 2 ± 2% of PM endocytosis in cells where endocytosis was blocked by dynamin inhibition with hydroxy‐dynasore (**Figure** **2**A,B) or first‐generation Dynasore (Figure S1F, Supporting Information). While endocytosis was inhibited even after 10 min in cells with dynamin inhibition, cells that repaired showed fourfold increased endocytosis by 10 min post injury (from 6.4% to 25.9%), with endocytosed WGA localizing in early endosomes (Figure 2B–D). Interestingly, similar transient attenuation of endocytosis was observed following mechanical scrape injury in transferrin‐labeled cells, that subsequently increased by 10‐min post‐injury (Figure S3A,B, Supporting Information). In this respect, though uninjured cells showed robust clathrin and caveolin‐mediated endocytosis, mechanical injury suppressed both these endocytic processes to levels comparable to dynamin‐inhibited cells within 30‐s post‐injury. Moreover, injured cells required ≈3 min post‐injury for their rate of endocytosis (marked by mobile fraction of vesicles) to approach levels seen in uninjured cells (Figure 2E–H). This suggested that cells may not require acute activation of endocytosis over the course of successful repair from focal PM injury. To assess the requirement of acute endocytosis in PM repair from focal injury, we used the above calcein labeling approach and monitored dye loss over the course of repair following PM injury. Control cells repaired efficiently, allowing minimal leakage of the calcein dye, while the dye leaked out fully from cells injured in absence of Ca^2+^, thus establishing the utility of this assay (Figure 2I,J). Cells where dynamin was inhibited acutely (30 min) by treatment with hydroxy‐dynasore, or dynasore, repaired similar to the control cells, however, chronic inhibition (24 h) of endocytosis by hydroxy‐dynasore caused failure of PM repair, which was reversed when cells were washed free of hydroxy‐dynasore for 2 h (Figure 2I,J; Figure S3C,D, Supporting Information). Quantification of injured cells that failed to repair, corroborated the finding that PM repair was blocked by the absence of extracellular Ca^2+^ and by chronic, but not by acute, inhibition of endocytosis (Figure 2I–K). Examining these findings in the context of endosome formation, we confirmed that chronic dynamin inhibition (via hydroxy‐dynasore) depleted, or attenuated early endosome formation, an effect that can be reversed with inhibitor washout (Figure S4, Supporting Information).

As focal PM injury triggered a delayed (>3 min post injury) increase in endocytosis and since depletion of endosomes by chronic inhibition of endocytosis impaired repair, we next examined if delayed (post PM resealing) activation of endocytosis supports endosome formation to facilitate PM repair. For this, we injured the PM and incubated injured cells for 30‐min in the absence (control), or presence of hydroxy‐dynasore to acutely block endocytosis during‐ and following PM repair. These cells were then injured for a second time and their ability to repair was assessed using the calcein leakage approach (Figure 2L,O). As is previously shown,^[^ ^29^ ^]^ control cells repaired more efficiently from the second bout of injury, exhibiting lower calcein leak following PM injury as compared to the initial injury (Figure 2M,N vs Figure 2I,J blue calcein kinetic traces). Inhibition of endocytosis following an initial injury, however, compromised the ability of the cell to repair from a subsequent injury (Figure 2M,O). Together, the above results identified that the injury‐triggered delayed (not acute) endocytosis is required for repair of focal PM injury, and depleting cells of early endosomes compromise the repair of focal PM injury.

### 2.3 PM Injury Causes Rapid Ca^2+^‐Triggered Exocytosis of Early Endosomes

**Figure 3:**
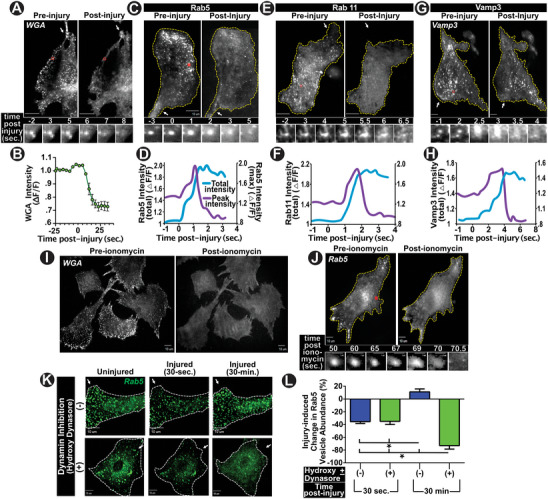
Early endosomes undergo Ca^2+^‐regulated exocytosis upon PM injury. Confocal image of early endosomes labeled for 10‐min with WGA^AF^ prior to or following focal laser injury (white arrow). Red‐box shows the region zoomed in images below showing single endosome undergoing injury‐triggered exocytosis. B) Plot showing kinetics of change in WGA^AF^ fluorescence of 40 select WGA‐labeled vesicles in (A). C,E,G) TIRF images of cells expressing early‐endosome markers Rab5, Rab11, or VAMP3 prior to or following focal laser injury (white arrow). Zoomed image series in the bottom panels shows Individual vesicles undergoing injury‐triggered exocytosis. D,F,H) Plot showing kinetics of injury‐triggered change in fluorescence of individual early endosome shown in the zoom. Traces show intensity of the brightest pixel (Peak intensity) and sum of fluorescence of all pixels in the zoomed image (Total intensity). Note the rise in peak intensity as the vesicle is pulled closer to the PM for fusion and loss of it as the fluorescence diffuses in the PM. I) Confocal images of WGA^F488^‐labeled early endosomes in cells prior to and 2 min following stimulation with calcium ionophore. J) TIRF image of Rab5‐GFP‐labeled early endosomes (and zoomed in single endosome in bottom image series) prior to and 1.5 min following calcium ionomycin treatment as in I. Note the full fusion of all endosomes and dispersion of vesicular fluorescence into the PM in both (I) and (J). K) Confocal images of Rab5‐GFP‐transfected cells subjected to focal laser injury to visualize the fate of early endosome upon focal injury with (+) (bottom panels) or without (‐) (top panels) the block in endocytosis by the dynamin inhibitor. L) Plot showing change in the proportion of Rab5‐GFP labeled early endosomes from before to after focal injury of cells that were either not treated (blue) or treated (green) with dynamin inhibitor, followed by images at 30 s or 30 min post‐injury; (n= 10‐20 cells per condition, 2 replicates). Note that endosome depletion by injury‐triggered exocytosis is replenished when compared to cells where dynamin is not inhibited for 30 min, but 30 min of dynamin inhibition causes even greater loss of these endosomes; All *p ≤ 0.01 as assessed by 1‐way ANOVA. Scale bars all 10 µm. White arrows depict site of focal laser injury. All data indicate mean + SEM.

While pursuing the above experiments, we serendipitously observed that focal PM injury caused the fluorescent WGA‐labeled endosomes to rapidly undergo exocytosis (**Figure** **3**A,B). While late endosomes/lysosomes are known to undergo injury‐triggered exocytosis, WGA‐labeled structures do not co‐localize with LAMP1 and instead co‐localize with the early endosome marker Rab5 (Figure 2C,D). This suggested that PM injury is triggering exocytosis of early endosomes. To confirm this, we expressed early endosomes marker Rab5 tagged with GFP (Rab5‐GFP) in cells and imaged them by total internal reflection fluorescence microscopy (TIRFM) to monitor response of these early endosomes to PM injury. This showed a robust and rapid injury‐triggered disappearance of these early endosomes following PM injury (Figure 3C; Video S1, Supporting Information). To determine if this represents exocytosis or injury‐triggered movement of the Rab5 vesicles, we used the TIRFM‐based assay we have previously described, to distinguish vesicle exocytosis from movement or lysis.^[^ ^30^ ^]^ Monitoring Rab5‐GFP fluorescence of individual endosomes as they disappeared following injury showed increase in the peak and total intensity as the endosome disappeared, followed by decrease in peak intensity while the total intensity remained elevated (Figure 3C,D). This response occurs when the vesicle fuses with the PM, delivers its membrane cargo (Rab5‐GFP) to the PM, allowing exponentially greater excitation of GFP by TIRF illumination resulting in increased total and peak intensity. This is followed by a decrease in peak intensity as the Rab5‐GFP diffuses in the PM, away from the site of fusion, yet the total intensity remains elevated due to efficient illumination of all Rab5‐GFP now present within the PM.^[^ ^30^ ^]^ To further establish this injury‐triggered exocytic fate of early endosomes, we examined additional early endosome markers – Rab11 and Vesicle associated Membrane Protein 3 (VAMP3). Similar to Rab5, early endosomes marked by these membrane proteins also rapidly disappeared following PM injury (Figure 3E,G). Here, once again, the vesicle exocytosis assay established the disappearance of Rab11 and VAMP3 was due to exocytosis and delivery of these membrane cargoes to the PM (Figure 3F,H).

While recycling endosomes constitutively fuse with the PM, they have not been shown to undergo regulated exocytosis. To examine if injury‐induced early endosome exocytosis represents Ca^2+^‐regulated exocytosis, we used calcium ionophore (ionomycin) to raise cellular calcium without injuring the PM, and found this triggered exocytosis of both early endosome markers examined – fluorescent WGA and Rab5‐GFP (Figure 3I,J; Video S2, Supporting Information). This observation raised the possibility that the accelerated endocytosis we observed following repair of focal PM injury, may facilitate replenishment of the pool of early endosomes that are lost due to Ca^2+^‐triggered exocytosis upon initial PM injury. To examine this, we monitored the fate of Rab5‐GFP‐labeled early endosomes over the course of successive PM injuries in control cells, and upon inhibition of endocytosis after an initial round of focal PM injury. Within 30 s of PM injury ≈40% of Rab5‐labeled endosomes exocytosed in both groups of cells (Figure 3K,L). When allowed to recover for the next 30 min, as expected, only the control cells restored the lost early endosomes, to pre‐injury levels (Figure 3K,L). This observation led us to hypothesize that the poor repair of the second round of focal PM injury observed above (Figure 2M–O), is caused by the lack of injury‐triggered early endosome exocytosis in cells where endocytosis was inhibited after the initial round of injury, preventing replenishment of their early endosomes after initial injury.

### 2.4 Rab5 Regulates Ca^2+^‐Triggered Early Endosome Exocytosis and PM Repair

**Figure 4:**
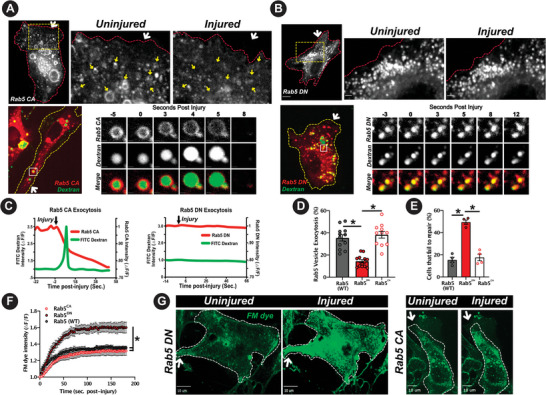
Rab5 facilitates focal injury‐triggered early endosome exocytosis and PM repair. A.C. TIRF (top panel) or confocal (bottom panel) microscopy images of cells expressing A) Rab5^CA^‐mCherry‐transfected and B) Rab5^DN^‐mCherry. Zoomed images show the yellow‐boxed region prior to or following injury (yellow arrows mark endosomes that exocytosed). Endosomes in bottom panel of cells were also labeled with FITC‐dextran endocytosis. Image series depict individual dual‐labeled endosomes at indicated times post injury. C) Plots showing kinetics of FITC dextran and mCherry‐tagged Rab5^CA^ and Rab5^DN^ fluorescence prior to and following PM injury. D) Plot showing the proportion of Rab5+‐endosome present prior to injury that underwent exocytosis upon injury. E–G) Plots and images of cells expressing WT and mutant Rab5 that were injured in presence of FM dye. E) Plot showing proportion of cells that failed to repair from focal injury, F) Plot of FM‐dye entry kinetics in injured cells, and G) Images of cells expressing Rab5^DN^‐mCherry (left) and Rab5^CA^‐mCherry (right) cells prior to and 3‐min post focal PM injury (white arrow). Data represents n=10‐15 cells for A, B, and n = 10‐20 cells for E‐G, with 4 replicates for repair assay. *P<0.05 for denoted comparisons. Scale bars (Whole cell images = 10 µm), (A, C top: 2 µm, bottom: 1 µm).). Multiple comparison of Rab5+ endosome exocytosis (D), and proportion of cells that fail to repair (E) assessed via 1‐way ANOVA. Kinetics analyses (F) performed via one‐way repeated‐measures ANOVA with post‐hoc t‐tests and Bonferroni correction for each timepoint. All data indicate mean + SEM.

To test our hypothesis that early endosome exocytosis is required for PM repair, we assessed whether Rab5 – a well‐established regulator of early endosome fusion, also regulates their exocytosis. To analyze this, we expressed mCherry‐tagged constitutively active Rab5 (Rab5^CA^) mutant or dominant negative Rab5 (Rab5^DN^) mutant in cells, and briefly fed these cells fluorescein‐labeled dextran to simultaneously label the membrane (Rab5) and lumen (dextran) of these early endosomes. Monitoring these dual‐labeled endosomes showed Rab5CA expression caused these early endosomes to be enlarged (**Figure** **4**A).^[^ ^31^ ^]^ Following PM injury, while Rab5CA endosomes underwent rapid exocytosis and released both (luminal and membrane) cargoes, endosomes labeled with Rab5^DN^ did not release either the luminal or the membrane marker, demonstrating the inability of these vesicles to undergo Ca^2+^‐triggered exocytosis (Figure 4A–D). This established Ca^2+^‐regulated early endosomes exocytosis as a novel role of Rab5 and provided a direct tool to examine the requirement of Ca^2+^‐triggered early endosome exocytosis for PM repair. Upon a single bout of focal PM injury of cells expressing WT Rab5, Rab5^CA^ and Rab5^DN^, cells expressing Rab5 and Rab5^CA^ repaired efficiently while those expressing Rab5^DN^ took in greater amount of FM dye and fewer of them managed to repair (Figure 4E–G). This demonstrated that endocytosis mediates PM repair from focal injury by generating early endosomes that undergo exocytosis to repair the injured PM, lack of which in Rab5DN expressing cells compromises PM repair even from a single bout of focal injury.

**Figure 5:**
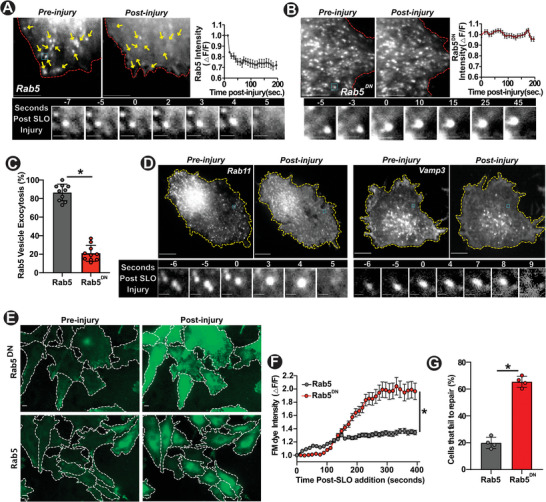
Rab5 and early endosome exocytosis are required to repair diffuse PM injury. A,B) Top panel shows confocal images of cells expressing A. Rab5^WT^‐mCherry and B. Rab5^DN^‐mCherry prior to or following SLO‐injury. Yellow arrows indicate individual endosomes that underwent exocytosis upon PM injury. Bottom panel shows zoomed image series showing individual vesicles post SLO‐injury in the cyan‐box portion from the top panels, and plots show the kinetics of change in Rab5‐mCherry fluorescence intensity of these endosomes. C) Proportion of Rab5‐labeled endosomes in uninjured cells that underwent exocytosis upon SLO injury of the PM. D) Images showing Rab11‐GFP (left) and Vamp3‐mCherry (right) expressing cells imaged as above (A,B), pre/post SLO injury. E) Images and F) plot showing FM dye entry kinetics in cells expressing Rab5^DN^‐mCherry and WT Rab5‐mCherry prior to following SLO injury of the PM. G) Proportion of cells that failed to repair from SLO injury (n=10 cells/condition, 3 replicates in panel C, n=30‐40 cells/condition, 4 replicates in panel G. Post injury images were acquired 7–10 min post SLO addition; Scale bars ‐ A, B top: 5 µm, bottom: 1 µm, D top, E: 10 µm, D, bottom: 1 µm. Paired data shown in panels C, G are assessed via independent t‐test and kinetics analyses in F by 1‐way repeated‐measures ANOVA with post‐hoc t‐tests and Bonferroni correction for each timepoint. All data indicate mean + SEM.

With the differential role of injury‐triggered endocytosis for PM repair of focal and diffuse injury, we next examined if injury‐triggered exocytosis also serves different roles in the repair of these distinct modes of PM injury. First, we monitored the Rab5‐labeled early endosomes upon SLO injury, and found that like focal injury, SLO injury triggered rapid early endosome exocytosis as well (Figure **5**A,C). Similar to focal injury, Rab5^DN^ expression inhibited the ability of early endosomes to undergo exocytosis in response to diffuse (SLO) injury (Figure 5B,C). Further, use of other markers – Rab11 and VAMP3, confirmed SLO injury‐induced exocytosis of early endosomes (Figure 5D). These results established that injury‐triggered exocytosis of early endosomes and Rab5‐mediated regulation of this exocytic response is conserved between focal and diffuse PM injury. Next, we examined if early endosome exocytosis is also required for the repair of SLO‐injured PM. This showed that inhibition of early endosome exocytosis by Rab5^DN^ prevented repair of the SLO injured cells, causing increased FM dye entry in Rab5^DN^ expressing cells and leading to fewer cells repairing from SLO injury (Figure 5E–G). These results identified that while injury‐triggered endocytosis is differentially required for the repair of cells from focal and diffuse PM injury, requirement of injury‐triggered exocytosis for PM repair is conserved between focal and diffuse PM injury.

## 3 Discussion

The PM serves as the site for all cellular communication between the cytoplasm and the extracellular environment, leading to its constant exposure to damaging mechanical and chemical signals and stresses.^[^ ^2^, ^3^, ^5^ ^]^ Repair of these injuries requires rapid orchestration of various cellular signals and organelle responses to ensure effective repair of the PM.^[^ ^2^ ^]^ These include PM trafficking by shedding, endocytosis, pinocytosis, and lysosome exocytosis, mitochondrial ROS‐activated actin‐mediated wound closure and ER‐mediated buffering of Ca^2+^ influx.^[^ ^3^, ^7^, ^10^, ^14^, ^16^, ^24^, ^32^ ^]^ Our study adds early endosomes as a novel cellular compartment that is required for repair of the injured PM. We find early endosomes undergo rapid Ca^2+^‐triggered exocytosis in response to large focal injury as well as nanoscale PFT injury, and this is required for repairing these forms of PM wounds. Previous studies have demonstrated lysosomes are the mammalian cell compartment that undergoes Ca^2+^‐triggered exocytosis to repair PM injury.^[^ ^10^, ^33^ ^]^ This is analogous to the Ca^2+^‐triggered exocytosis of yolk and or cortical granules in oocytes thought to provide the endomembrane to patch the lost PM.^[^ ^34^, ^35^ ^]^ However, subsequent studies have identified that injury‐triggered lysosome exocytosis is required for releasing lysosomal enzymes such as ASM that enable endocytosis to help clear the damage in the PM.^[^ ^21^, ^22^, ^23^, ^24^ ^]^

We find that while diffuse PFT injury triggers endocytosis over the course of PMR, large focal injury acutely freezes endocytic activity during PMR, reactivating it once the wound is repaired (Figures 1 and 2). What causes endocytosis to freeze upon focal injury and be reactivated after apparent wound resealing, remains elusive. ASM released by exocytic lysosomes leading to hydrolysis of PM sphingomyelin into ceramide, offers a potential mechanism for late‐onset activation of endocytosis in focally injured cells. The relevance of this acute activation of endocytosis by PFT injury is demonstrated by poor repair of SLO‐injured cells when endocytosis is inhibited (Figure 1). This may be due to the proposed requirement of endocytosis to remove the toxin from the injured PM.^[^ ^24^ ^]^ A similar requirement for dynamin‐dependent endocytosis in PM repair from diffuse injury has been described for perforin‐mediated PM injury.^[^ ^36^ ^]^ However, endocytosis does not help remove other PFT subtypes (Listerolysin O) from injured PM.^[^ ^37^ ^]^ As PFTs can be removed by membrane shedding, removal of toxin pores from the PM can be accomplished via multiple mechanisms.^[^ ^38^, ^39^, ^40^ ^]^ In support of this view a recent study identified that most of the PM‐bound PFTs (Perfringolysin O, SLO) are removed by way of membrane shedding.^[^ ^40^ ^]^ What then, could be the role of endocytosis? It is suggested that PM endocytosis may help restore lipid homeostasis of the repaired PM.^[^ ^37^ ^]^ Thus, endocytosis may contribute to PFT pore removal or play a homeostatic role in the repaired cell. Such a homeostatic role for endocytosis is exemplified in our findings that replenishment of early endosomes that were lost due to exocytosis in repair of focal PM injury, was reliant upon post‐repair endocytosis (Figure 2). This homeostatic role of early endosome replenishment is critical for cell survival if there were to be another tear in the newly repaired PM (Figure 3). This demonstrates how diffuse versus focal PM injury utilizes the same compartment (early endosomes) for different membrane trafficking roles to support PM repair.

The requirement of early endosomes to undergo Ca^2+^‐triggered exocytosis for PMR is not limited to focal injury, as its inhibition compromised PM repair from focal and diffuse injury alike (Figures 2 and 5). Its ubiquity raises the question‐ what role does injury‐triggered early endosome exocytosis play in PM repair? As lysosome exocytosis supports PMR by promoting endocytosis, it raises the possibility that early endosomes could serve as the endomembrane reservoir for the mammalian cell PM. To qualify as a suitable membrane reservoir, it must be delivered rapidly (10–30 s);^[^ ^5^ ^]^ support repair of small and large wounds, and be rapidly replenished following its use for PMR. Early endosomes fulfill all these requirements, as they are a large intracellular membrane reservoir that is replenished quickly by PM endocytosis, and we show that they respond to PM injury by exocytosing within seconds of injury (Figure 3). Such a role of early endosomes is further supported by the fact that blocking their exocytosis compromises PMR (Figure 4). However, unlike the patch formation by cortical granules in oocytes,^[^ ^35^ ^]^ we did not observe any injury‐triggered homotypic fusion of early endosomes, nor did the endosomes migrate to the site of injury to fuse in a patch‐like manner. Instead, rapidity of their exocytosis was due to fusion at the location they were at the time of injury (Figure 4). Such a mode of addition of these endomembrane, supports the PMR model wherein the added membrane helps reduce PM tension, allowing for the wound to constrict (by annexins and other proteins) and close.

Our analysis uncovered a novel role of Rab5 as a regulator of PMR by facilitating early endosome exocytosis (Figure 3). A recent study independently validated focal, injury‐triggered exocytosis of early endosomes under the control of the SNARE VAMP2, which enables PM repair in endothelial cells.^[^ ^41^ ^]^ This is distinct from our prior studies using the SNARE VAMP8 to label early endosomes, that did not exhibit calcium‐triggered exocytosis following stimulation with calcium ionophore.^[^ ^10^, ^33^ ^]^ In this study we show that blocking early endosome exocytosis (by Rab5 inhibition) inhibits PMR not only from focal, but also diffuse PFT injury. This indicates that the role of early endosomes as an endomembrane reservoir is required for PM repair from both focal and diffuse injury (Figures 4 and 5). A role of Rab5 in PMR is supported by a previous study using PFT injury of the intestinal epithelium of *C. elegans* that showed that lack of Rab5 (and Rab11) suppressed PFT removal from the intestinal epithelium, including shedding of epithelial microvillius.^[^ ^42^ ^]^ However, such a role of Rab5 was noted over a slower timescale (0.5 to 24‐h) post injury. The roles of Rab5 and 11 in slow repair of *C. elegans* intestinal epithelium and of rapid PMR repair observed here in mammalian cells, requires further examination. However, the need for a faster activation of dynamin‐dependent endocytosis in mammalian cells is demonstrated by its requirement for repair of focal injury to muscle fibers.^[^ ^43^, ^44^ ^]^ This process involves intracellular accumulation of PM‐derived vesicles, which contain muscle cell membrane repair protein dysferlin. Subsequent fusion of these dysferlin vesicles assists in repair of both injured muscle cells and fibers.^[^ ^43^, ^44^, ^45^ ^]^ Such a model aligns with our observations here and extends it beyond single cells to multinucleated myofibers. Future studies are needed to examine the biochemical regulators and the timing of the mutually complementary use of endosomes for different forms of PM injury. Further, our study shows existence of PMR processes that are tailored to the nature (diffuse or focal) injury to the PM and identifies early endosomes as a unique organelle that is able to support both forms of PMR through its dual ability to function as an endocytic and an exocytic membrane reservoir.

## 4 Conclusion

We demonstrate that repair of PM injury by PFTs but not by focal injury activates endocytosis, and this is required for the repair of PFT injury. However, we find PFT and focal PM injury both activate early endosome exocytosis that is Ca^2+^‐triggered and regulated by Rab5 activity. Further, injury‐triggered exocytosis of early endosomes is required for repair from both forms of PM injury identifying a novel essential function of early endosomes in cells.

## 5 Experimental Section

### Cell Culture and Treatment

C2C12 cells (ATCC, CRL‐1772) were cultured in high‐glucose DMEM supplemented with 10% FBS and 100 ug mL^−1^ penicillin and 100 ug mL^−1^ streptomycin and maintained at 37 °C and 5% C0_2_. Cells were seeded on coverslip and transfected using Lipofectamine 2000 (Invitrogen, Waltham MA) for 6 h, followed by 12–48‐h recovery period in full growth media prior to live‐cell imaging. Plasmids used were obtained from Addgene. For inhibiting endocytosis, cells grown on coverslip were washed 3× with calcium‐free PBS, and incubated for 30 min in serum‐free media supplemented with 30 µm Hydroxy‐Dynasore in DMSO (Dyngo‐4a, MCE, hereafter referred to as Hydroxy‐Dynasore), 80 µm first‐generation Dynasore (Dynasore) or equivalent amount of DMSO (Control). For Calcein labeling, washed cells were labeled using 1 µm working concentration of Calcein red/orange in a 37 °C CO_2_ incubator for 30 min. Cells in above treatments were washed 3–5× with 1× PBS and transferred to cell imaging media (CIM) or CIM with DMSO (control), 30 µm Hydroxy‐Dynasore, or 80 µm Dynasore, for imaging PM repair (reagent list – Table S1, Supporting Information).

### Diffuse PM Injury with PFT Streptolysin O

Cells grown on coverslips (60–70% confluence) were washed twice with cell image media (CIM: HBSS with 10 mM HEPES, pH 7.4) and maintained in CIM in Tokai Hit microscopy stage‐top ZILCS incubator (Tokai Hit Co., Fujinomiya‐shi, Japan) at 37 °C for the duration of experiment. PFT injury was conducted in CIM (with or without supplementation with 2 mm calcium). Stock SLO (Sigma #S5265) was solubilized in water to a concentration of 157068 Units per mL, and single‐use aliquots stored at −20 degrees, as per manufacturer's instructions. Working solutions of SLO were thawed, titrated to varying concentrations (ranging from 200 U mL^−1^–1000 U mL^−1^) with prior activation in 20 mm DTT for 15‐min at 37 °C. Activated working solutions were then added to the cell imaging chamber, and cells were imaged via time‐lapse microscopy using an IX81 Olympus spinning disk confocal microscope (Olympus America, Center Valley, PA, USA), with a 40X/ or 60X/1.45NA oil objective. 5–10 fields of view, containing ≈4–10 cells each were imaged over the injury and repair period at 1 frame/sec. for diffuse PFT dosage experiments by automated stage visiting for each position to enable repeated imaging of the same cells throughout various labeling approaches outlined below.

### Repair Kinetics Following Diffuse PFT Injury

Cells were prepared as above using CIM supplemented with membrane impermeable FM 1–43 fluorescent dye (working concentration of 5 ug mL^−1^) (Invitrogen, #T3163). Kinetics of cell repair of diffuse PFT injury was obtained by tracking the cellular influx of FM dye over time (10 min) expressed as the change in fluorescence intensity relative to baseline (i.e., ΔF/F, where F is the baseline fluorescence intensity prior to injury). Cells were deemed to be injured if cellular fluorescence exceeded 20% above baseline (indicative of dye influx). At the conclusion of the 10‐min injury and repair period, success or failed repair was determined by removing the SLO‐containing CIM and replacing with fresh CIM supplemented with 2 ug mL^−1^ Propidium Iodide (PI). Labeling was allowed to proceed for 5–10 min, followed by re‐imaging all saved cell fields. Cells failed to repair if they were PI+. For endocytosis blocking studies with diffuse injury, Dynasore compound binds and co‐transports free FM 1–43 dye into cells, thus precluding the use of FM 1–43 dye for these experiments. Thus, this study turned to membrane‐permeant Calcein red‐orange (Invitrogen, Waltham MA). With this approach, following PM injury, cells leak their calcein dye contents until the membrane was resealed – thus, loss of Calcein fluorescence, tracked over time, served as another viable measure of membrane repair kinetics. Kinetics of cell repair of diffuse PFT injury was obtained by tracking the cellular loss of Calcein dye, or the change in fluorescence intensity relative to baseline (i.e., ΔF/F), upon addition of activated SLO (at 400 U mL^−1^ working concentration), as described above.

### Assessment of Endocytosis upon Diffuse PFT Injury

Assessment of the effects of diffuse PFT on bulk membrane endocytosis was completed as previously described.^[^ ^26^ ^]^ Briefly, cell plasma membranes (at density of ≈70% confluency) were labeled with Alexa Fluor 488–conjugated WGA (3 µg mL^−1^) for 2 min at 37 °C, or ice‐cold Alexa Fluor 488‐conjugated Transferrin (Tfn) (25 ug mL^−1^) for 5 min. After washing the excess label with CIM, cells were left untreated/uninjured or PFT‐injured with SLO as above, and imaged simultaneously in widefield and confocal modes. Cell PM repair and WGA/Tfn endocytosis was allowed, and at different time points following observable PFT injury, bromophenol blue (BPB) was injected into the imaging chamber (final concentration of 5 mm) to quench non‐internalized WGA/Tfn at the cell surface. This quenching paradigm spares internalized fluorescent membrane, and thus allowed sto determine the fraction of membrane internalized after the above‐mentioned endocytosis periods. To assess extent of this membrane endocytosis, following back‐ ground correction, the average postquench fluorescence of each cell was divided by its initial prequench fluorescence, and normalized to the fraction of internalized membrane assessed after immediate quenching (0‐min endocytosis) using image analysis software (SlideBook 6.0, 3i intelligent Imaging Innovations, Inc. Denver, CO, USA). To visualize and track the effect of diffuse PFT injury on specific endocytic pathways, cells were transfected with mRFP or GFP‐tagged caveolin‐1 (Caveolae) or clathrin (Clathrin coated structures ‐CCS), and imaged as described previously to monitor their motility/disappearance.^[^ ^46^ ^]^

Cells were imaged in CIM with a 60×/1.45 NA oil objective as described above, using an Olympus IX81 microscope equipped using a 488 and 560 nm confocal diode laser (Cobolt), at the membrane‐coverslip interface. Transfected cells were subjected to diffuse PFT injury and imaged at 1 frame/sec. over a 7–10‐min injury‐repair period. To visualize caveolae and CCS turnover reflective of endocytic activity, select cell regions containing membrane‐proximal caveolae and CCSs were compared at the onset of SLO injury and 2‐min after injury, to observe caveolae or CCS mobility (indicated by the lack of colocalization of caveolae and CCSs between their initial position and final position at timepoint 2). Lastly, for visual representation of clathrin and caveolin vesicle mobility/turnover in a single image, timelapse kymographs were constructed from embedded horizontal lines within select cells (via SlideBook 6.0 Imaging Software – Intelligent Imaging Innovations, Inc.). Time was represented by the y‐axis or height of the kymograph chart, while the spatial distribution of vesicles along the cell‐embedded lines, was demonstrated on the x‐axis or width of the kymograph chart. Caveolin‐ and Clathrin‐transfected cells (and non‐transfected) were also subject to the same diffuse PFT injury, after WGA‐labeling, as outlined above, to assess the contribution of caveolin and calthrin overexpression to PM endocytosis upon diffuse PFT injury.

### Repair Kinetics Following Focal Laser Injury

Cells were subjected to focal mechanical PM injury via pulsed laser ablation as described previously.^[^ ^26^ ^]^ Briefly, cells were grown and prepared as described above, and microscope‐mounted cells were laser injured in CIM supplemented with FM 1–43 dye (5 ug mL^−1^) at 37 °C in the stage‐top ZILCS incubator (Tokai Hit Co.). To achieve focal injury, a 1‐ to 5‐µm^2^ area of plasma membrane was mechanically disrupted via pulsed laser induce cavitation for <10 ms with a pulsed laser (Ablate!, 3i Intelligent Imaging Innovations, Inc. Denver, CO, USA), and cells were imaged at 1 frame/sec. with a 40x or 60×/1.45 NA oil objective with a 488 or 564 nm diode laser (Cobolt). All fields were saved in microscope operation software (SlideBook 6.0) to enable return to these injured cells for downstream labeling or post‐injury/repair assessments. Kinetics of cell repair of these focal injuries was determined as for PFT injury above – tracking the cellular influx of FM dye over time (3 min) expressed as the change in fluorescence intensity relative to baseline (i.e., ΔF/F, where F was the baseline fluorescence intensity prior to injury). Cells were deemed to be injured if cellular fluorescence exceeded 20% above baseline (indicative of dye influx), and cells that failed to repair were determined by the unabated increase in FM 1–43 dye fluorescence over the 3 min following PM injury.

For assessing the effects of chronic endocytosis inhibition and repeat injury on PM repair two different respective approaches were taken. To assess the effects of chronic endocytosis inhibition, cells were incubated in 30 µm Hydroxy‐Dynasore (or equivalent DMSO concentration‐ 0.3%) for 24 h (in non‐transfected and Rab5‐mRFP‐transfected cells). For chronic dynamin inhibition with washout conditions, cells were incubated in 30 µm Hydroxy‐Dynasore for 24 h as above, with a 2‐h washout period (equivalent DMSO‐ 0.3%), followed by live‐cell assays (i.e., repair assays and Rab5 abundance assessments). To assess the effects of acute endocytosis inhibition, cells were pre‐loaded with Calcein red/orange dye as above, and with cell CellMask green (Invitrogen) in CIM supplemented with 1.5X working CellMask green, stained for 10‐min. at 37 °C at ambient C0_2_ with or without Dynamin inhibition (both Hydroxy‐Dynasore and Dynasore were used, and equivalent DMSO concentration). Use of CellMask aided in optimal focus on the PM for precise injury. Kinetics of cell repair and success of repair of focal laser injury, for Rab5 mutant experiments, was obtained by tracking the cellular influx of FM dye over time (3‐minpost‐injury), as for PFT injuries above. To assess PM repair upon repeat injury or endocytosis inhibition conditions, cells were imaged in both green and red channels, using green CellMask labeling to guide PM injury, and kinetics of cell repair was obtained by quantifying loss of Calcein dye (relative to baseline – ΔF/F) as for PFT injuries.

### Assessment of Endocytosis Following Focal Laser Injury‐Induced Endocytosis

To assess bulk PM endocytosis in response to focal PM injury (with or without endocytosis inhibition via Dynasore), cells were labeled with AF‐488‐conjugated WGA as for PFT injury experiments above, and injured as described previously.^[^ ^26^ ^]^ Briefly, WGA‐labeled cells were imaged using an IX81 Olympus microscope (Olympus America, Center Valley, PA, USA), with a 60X/1.45NA oil objective, equipped with a diode laser of 488 nm (Cobolt, Sweden) in both widefield and confocal modes, simultaneously. The imaged membrane‐labeled cells were subsequently allowed to endocytose their AF‐488‐labeled membranes for 0, 3, or 10 utes (all either following or absent‐of laser injury), at which time, 20 mm membrane impermeable bromophenol blue (BPB) was immediately diluted in the imaging chamber to a 5 mm working concentration, to quench non‐internalized fluorescent membrane for quantification of the proportion of membrane internalized as for PFT injury above.

To quantify caveolin and clathrin mobility with laser injury, 50 individual caveolin or clathrin puncta/vesicles were marked in each cell at the start of imaging. For analysis, each pre‐marked puncta/vesicle were assessed for each cell, with the rater blind to condition. Each vesicle was subsequently tracked manually using a strict scoring criterion. A vesicle (or component vesicle of a caveolin cluster) was deemed mobile if it either migrated laterally for a distance of >1.5 µm or moved axially such that it was absent from the imaging plane for 10 s or longer, or both. To avoid the pitfalls of measurement approximation and ensure exact measurement, pre‐selected puncta/vesicles were first bounded by a circle with a 1.5 µm radius (to provide lateral motility boundaries for each vesicle). A vesicle was marked as mobile and the timeframe at which it exhibited mobility was noted and binned into one of four‐time windows – mobile within 30 s of the commencement of image acquisition or within 30 seconds of injury, mobile within the first minute, first 2 min, and first 3 min. Quantification was a cumulative analysis, as a tracked vesicle that was mobile in the first 30 s of image acquisition or injury, was also marked as being mobile within the first minute and so on. The fraction of mobile vesicles (out of 50 for each cell) was quantified over the 3‐min time point (post‐injury or no‐injury ± Dynasore), per cell, with 5 cells assessed per experiment (3‐4 experimental repeats) as described previously.^[^ ^26^ ^]^

### Assessment of Clathrin‐Mediated Endocytosis Following PM Injury

To visualize and track the effect of injury on the clathrin‐mediated endocytic pathway, cells were transfected with GFP‐tagged clathrin, and imaged as described previously.^[^ ^46^ ^]^ To monitor endocytosis of clathrin‐coated structures – CCS, luster motility of CCS prior to‐ and following injury was quantified, as has been performed by others.^[^ ^47^ ^]^ CCS movement/dissapearance can also be caused by the disassembly of clathrin.^[^ ^48^ ^]^ Thus, transferrin labeling was made to use as an alternate strategy to monitor clathrin‐mediated endocytosis.

Briefly, cells were cultured in 10‐cm dishes to 70% confluency. At this time, cells were washed 3× in ice‐cold 1X PBS (calcium and magnesium‐free) and labeled for 5 min in ice‐cold CIM supplemented with 25 ug mL^−1^ Tfn^AF488^. Cells were injured either via SLO treatment (as described above *assessment of endocytosis upon diffuse PFT injury*) or scrape approach as described previously^[^ ^49^ ^]^ For mechanical injury assays, Tfn‐labeling solution was replaced by pre‐warmed (37 °C) CIM supplemented with 2 mg mL^−1^ lysine‐fixable TRITC dextran (Invitrogen), and cells were immediately scraped with a cell lifter (VWR #10062‐910) and collected in 15 mL falcon tubes protected from light. In injury‐free conditions, cells were detached from the culture plate (via 5–10 mm EDTA in 1x PBS), pelleted, and labeled in ice‐cold suspension. Cells were then given time to repair mechanical injury for 3 or 10 min, at which time, cells were immediately fixed in PFA, pelleted, and re‐suspended in CIM. Cells were subsequently deposited on glass coverslips and non‐internalized Tfn‐label was quenched with addition of BPB as described above (see *Assessment of endocytosis upon diffuse PFT injury*) and imaged via confocal microscopy. For non‐injury experiments, Tfn‐labeled cells in ice‐cold suspension were pelleted, and re‐suspended in pre‐warmed CIM (± Dynamin inhibition via Hydroxy Dynasore) and allowed to endocytose TFN‐label for 3‐ and 10‐min, followed by immediate PFA fixation, and imaged as described for injury experiments.

To assess extent of this membrane endocytosis, following background correction, the average postquench fluorescence of single cells across conditions was divided by single cell initial prequench fluorescence, and normalized to the fraction of internalized membrane assessed after immediate quenching (0‐min endocytosis – cells fixed and imaged immediately after Tfn‐labeling followed by BPB quenching) using image analysis software (SlideBook 6.0, 3i intelligent Imaging Innovations, Inc. Denver, CO, USA). Proportion of Tfn internalized for each timepoint and condition was obtained by dividing the cellular Tfn fluorescence intensity post‐quench, to the cellular Tfn fluorescence intensity immediately after initial PM labeling intensity (immediately post‐labeling, without an endocytosis period – %). To confirm Tfn internalization occurs largely within the clathrin pathway, clathrin DsRed‐transfected cells were labeled with Tfn^AF488^ under ice cold conditions as described above. Endocytosis was commenced upon switching the labeling solution to pre‐warmed CIM, and allowed to proceed for 3‐min, at which time cells were fixed and imaged as above. To quantify Tfn^AF488^ internalized within CCS, individual cells were analyzed using the Manders Colocalization Coefficient (MCC) that provides a measure of the fraction of one protein/probe that colocalizes with that of another (i.e., Tfn with clathrin). MCC was obtained via SlideBook software.

### Endosome Exocytosis Tracking Following PM Injury

To track potential exocytosis of labelled endosomes (WGA‐, Rab‐, Vamp‐labelled endosomes), following WGA internalization, FITC‐conjugated dextran endocytosis (for Rab mutant studies) and/or transfection, prepared cells were imaged via live‐cell TIRF and/or confocal settings as described previously.^[^ ^28^, ^50^, ^51^ ^]^ Briefly, for determination of WGA+ endosome exocytosis and Rab5, Rab11, and Vamp3 endosome PM‐fusion upon focal laser injury, all cells were imaged in TIRF. For TIRF imaging, the penetration depth was set at 150 nm and imaged as above, at a rate of 2 frames/sec to capture exocytosis events. Endosome vesicle exocytosis with the PM displays distinct characteristics upon entering the TIRF evanescence field – specifically an increase and sustained elevation of its total vesicle fluorescence, coupled with a sharp increase and decay of their peak/maximal fluorescence intensity.^[^ ^28^ ^]^ Thus, to verify exocytosis and full membrane fusion of these endosome compartments, individual labeled endosomes (n = 5‐10 per cell) were briefly assessed for these fluorescence characteristics in the TIRF field, following injury (focal mechanical laser ablation cavitation). The same approach was also used to determine the calcium stimulation of endosome fusion via Ionomycin treatment (10 µm ionomycin working concentration added to CIM supplemented with 4–8 mm Ca^2+^). However, due to the challenges and strict requirement that cells remain well‐adhered to the coverslip surface to maintain good‐quality TIRF field imaging, confocal imaging was preferred as a means to quantify endosome fusion, as it enabled higher throughput and increased cell numbers with a reliable imaging plane window in which to observe vesicle exocytosis following injury – an approach not practical for regular use of TIRF imaging. For confocal tracking of endosome exocytosis at the PM/coverslip interface, vesicle fusion was clearly observable as a sudden loss of vesicle fluorescence in the absence of any apparent cell retraction or z‐plane drift by the microscope – and was utilized for diffuse PFT injury tracking of endosome exocytosis for these same reasons. To quantify endosome fusion, total labeled puncta (Rab5 and all associated mutant Rab5s) were first manually counted pre‐injury and at the conclusion of the tracking period/post‐injury, and expressed as a percentage of total.

For confirmation of Rab5 mutant (Rab5 CA and Rab5 DN forms) endosome exocytosis upon injury, Rab5‐transfected cells (Rab5‐CA and Rab5‐DN mCherry) were incubated in serum‐free DMEM supplemented with 4 mg mL^−1^ FITC‐conjugated dextran (Invitrogen) for 1–2 h at 37 °C/5% C02. After this endocytosis period, cells were washed 3× with 1× PBS, and mounted for live‐cell imaging and focal laser injury as described above. To track endosome exocytosis in these conditions, cells were imaged in confocal mode at the cell membrane/coverslip interface z‐plane using the 60×/1.45NA oil objective, and 488 and 564 nm diode lasers, at a rate of 1 frame per second. Endosome exocytosis/or lack there‐of, was confirmed through assessment of individual endosomes for their concomitant increase in Rab5+ endosome size with increasing size and fluorescence intensity of its dextran cargo, followed by a rapid dispersion and drop in dextran and Rab5 fluorescence intensity. This was indicative of full fusion and expulsion of endosomal FITC dextran. Exocytosis was quantified as proportion of total endosomes that fused.

### Statistical Analysis

For PM injury and repair kinetics (Calcein Dye‐ and FM dye intensity kinetics), all generated curves were compared via mixed‐model ANOVA with analyses for interaction effects between the main effects of treatment condition and time. In the event of significant interaction, group differences in Calcein dye‐ or FM dye fluorescence intensity were assessed per time point via Holm‐Sidak test, and Huynh‐Feldt correction due to violation of sphericity. One‐way ANOVA was used to determine differences in the number of cells that failed to repair following injury (titrated SLO injury experiments, single injury experiments, Rab5 mutant experiments). Repeated‐measures ANOVA was used to assess for differences in PM endocytosis at different time points post‐injury across conditions, caveolae and CCS mobile fractions, and Rab5+ endosome abundance assessments at different times following injury. Comparisons between uninjured and injured cell PM endocytosis, ± Dynamin inhibition on proportion of cells that fail to repair, WGA compartment (Lamp1, Rab5) correlation measurements, Rab5 WT and mutant early endosome fusion proportion experiments, and proportion of failed repair analysis, were all calculated using independent samples *t* test. Similarly, independent samples *t* tests were used to calculate differences in PM endocytosis between non‐transfected and caveolin‐1 or clathrin‐transfected cells with SLO injury. One‐way ANOVA was used to assess differences in Rab5 fluorescence intensity measures of early endosome depletion in chronic dynamin inhibition experiments. For all statistical analysis, α level was set at *P* <0.05.

### Early Endosome Exocytosis Upon Focal PM Injury

Live cell imaging of a cell with Rab5‐GFP labeled early endosomes subjected to mechanical injury by a focal laser pulse‐induced cavitation bubble. Images were acquired in TIRF mode at the membrane‐coverslip interface (penetration depth of 150 nm), using a 488 nm laser excitation. Cell was imaged at 5 Hz and injury was induced at the lower left corner at timepoint 4.8 s. Video was played at 20 frames/second. Scale bar = 10 µm.

### Early Endosome Exocytosis Upon Calcium Influx

Live cell imaging of a cell with Rab5‐GFP labeled early endosomes subjected to cytosolic calcium increased by treatment with 10 µm ionomycin (in CIM with 6 mm Ca^2+^). Images were acquired in TIRF mode at the membrane‐coverslip interface (penetration depth of 150 nm), using a 488 nm laser excitation. Cell was imaged at 5 Hz for 2 min and video was played at 50 frames/second. Scale bar = 5 µm.

## Conflict of Interest

The authors declare no conflict of interest.

## Supporting information

Supporting Information

Supplemental Video 1

Supplemental Video 2

## Data Availability

The data that support the findings of this study are available from the corresponding author upon reasonable request.
