# Supplementary material for: Early Endosomes Undergo Calcium‐Triggered Exocytosis and Enable Repair of Diffuse and Focal Plasma Membrane Injury
Source: Adv Sci (Weinh). 2023 Sep 13;10(33):2300245. doi: 10.1002/advs.202300245 (PMC10667805; doi:10.1002/advs.202300245)
Supplement: Supplementary file 1 — Supporting Information [file ADVS-10-2300245-s002.pdf]

ADVANCED SCIENCE
Open Access

Supporting Information

for *Adv. Sci.*, DOI 10.1002/advs.202300245

Early Endosomes Undergo Calcium-Triggered Exocytosis and Enable Repair of Diffuse and Focal Plasma Membrane Injury

*Daniel C. Bittel and Jyoti K. Jaiswal**

Figure S1.

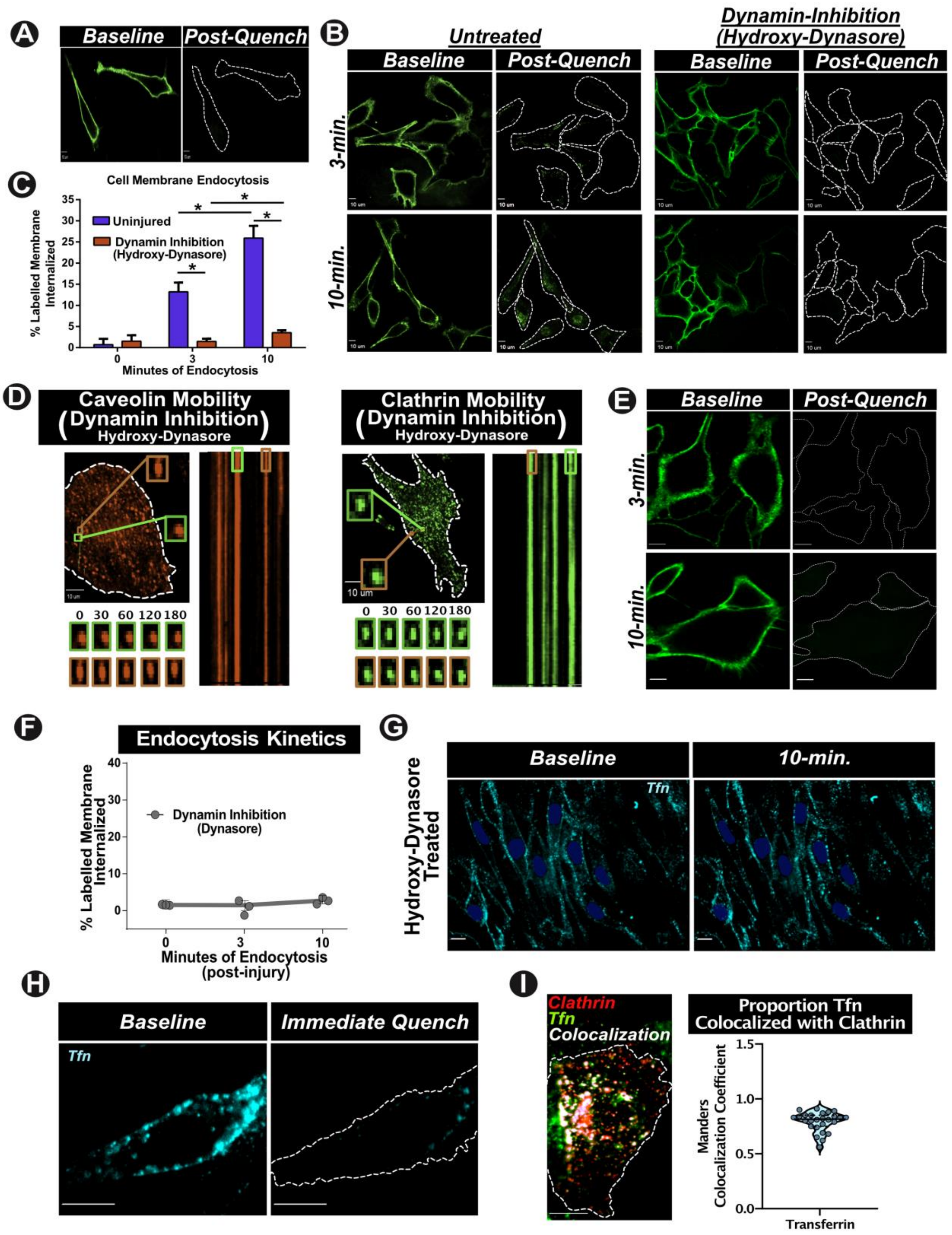

Figure S1. **Membrane turnover dynamics and its disruption can be reliably assessed via fluorescent labelling approaches.**

A-C. Images showing uninjured cells following surface labeling with WGA^{AF} to monitor bulk endocytosis. (A) Uninjured cells with immediate quenching (0 minutes of endocytosis). (B) Cells imaged immediately after PM labelling (left images) and again at 3-minutes (top panel) or 10-minutes (bottom panel) endocytosis upon quenching of surface WGA^{AF} with bromophenol blue (right images). Conditions shown are cells left untreated (DMSO-control) or dynamin-inhibited via Hydroxy-Dynasore. (C) Plot showing the kinetics of bulk endocytosis

in uninjured cells (DMSO-control - blue, dynamin-inhibited cells - red) (*P<0.05 vs. specified condition) (3min. - *Control vs. Dynamin-inhibited: p<0.01) (10-min. - *Control vs. Dynamin Inhibition: p<.001). **D.** Images and kymographs of Caveolin-mRFP and clathrin-GFP transfected cells subject to diffuse SLO injury with dynamin inhibition via hydroxy-dynasore. Orange and green inset boxes highlights single caveolin puncta or clathrin coated structures (CCS), that were tracked in kymograph images (vertical axis = time over 170 seconds). **E.** WGA^{AF}-labelled cells subject to diffuse SLO injury and dynamin inhibition (via hydroxy-dynasore) 3-minute (top) and 10-minutes (bottom) post-injury (quenched images right panel). **F.** Plot showing kinetics of bulk endocytosis in uninjured cells (using WGA^{AF}-labelling and BPB quenching approach) subject to dynamin inhibition with 1st generation Dynasore treatment. **G.** Uninjured Tfn^{AF488}-labelled cells subject to hydoryx-dynasore treatment. Images obtained immediately post- and 10 minutes-post labelling, depicting lack of internalization. **H.** Uninjured Tfn^{AF488}-labelled cell immediately after PM labelling (baseline) followed by immediate quenching of non-internalized fluorescence (immediate quench) – depicting the minimal Tfn internalized at time = “0 minutes of endocytosis”. **I.** Left- Representative confocal image of a cell transfected with Clathrin-DsRed and PM-labelled with Tfn^{AF488}, depicting considerable colocalization after 3-minutes of endocytosis. Right – Manders Colocalization Coefficient plot demonstrating ~80% of internalized labelled Tfn colocalizes with clathrin.

Figure S2.

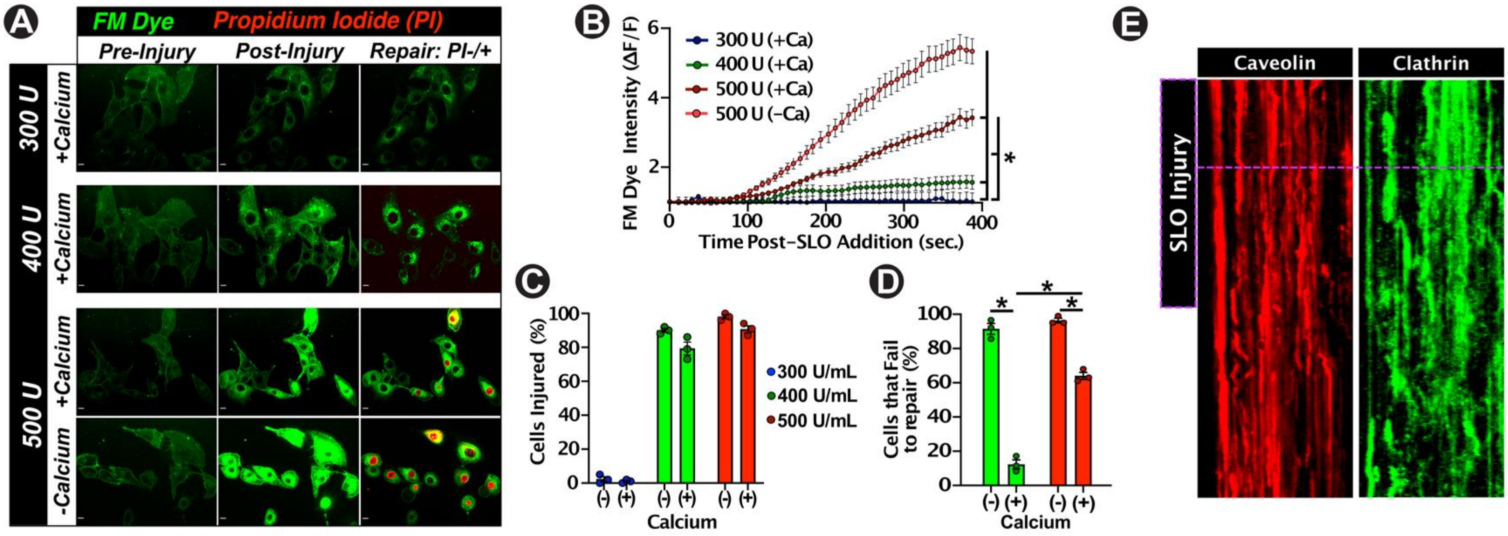

<u>Figure S2.</u> **Diffuse PM injury and repair induces bulk PM endocytosis**.
A. Images of cells diffusely injured with stated doses of SLO in the presence (+) or absence (-) of calcium. Increasing FM-dye fluorescence indicates greater dye entry following PM injury and nuclear propidium iodide (PI) staining marks cells that failed to repair and hence exclude PI dye entry. **B.** Plot showing kinetics of FM dye entry into cells following SLO-indued membrane injury *P<0.05 vs. indicated conditions by mixed-model ANOVA with analyses for interaction effects between treatment condition and time. **C.** Quantification of proportion of cells (shown in panel A) that were injured (FM dye labeled); presence or absence of Ca^{2+} did not alter the ability of SLO to injure cells *P>0.05). **D.** Quantification of proportion of inured cells (in panel A) that failed to repair (were labeled with propidium iodide). (*p<0.01). Data in (B-D) represent n = 40-50 cells per condition, with 3 experimental replicates. **E.** Kymographs for caveolar and clathrin endocytosis prior to and following SLO addition (purple line). *P<0.05. Kinetics analyses (B) performed via 1-way repeated-measures ANOVA with post-hoc t-tests and Bonferroni correction. Differences in cell repair and endocytosis determined via 1-way ANOVA for multiple comparisons (C, D). Scale bars (A = 10 µm). Data indicate mean ± SEM.

Figure S3.

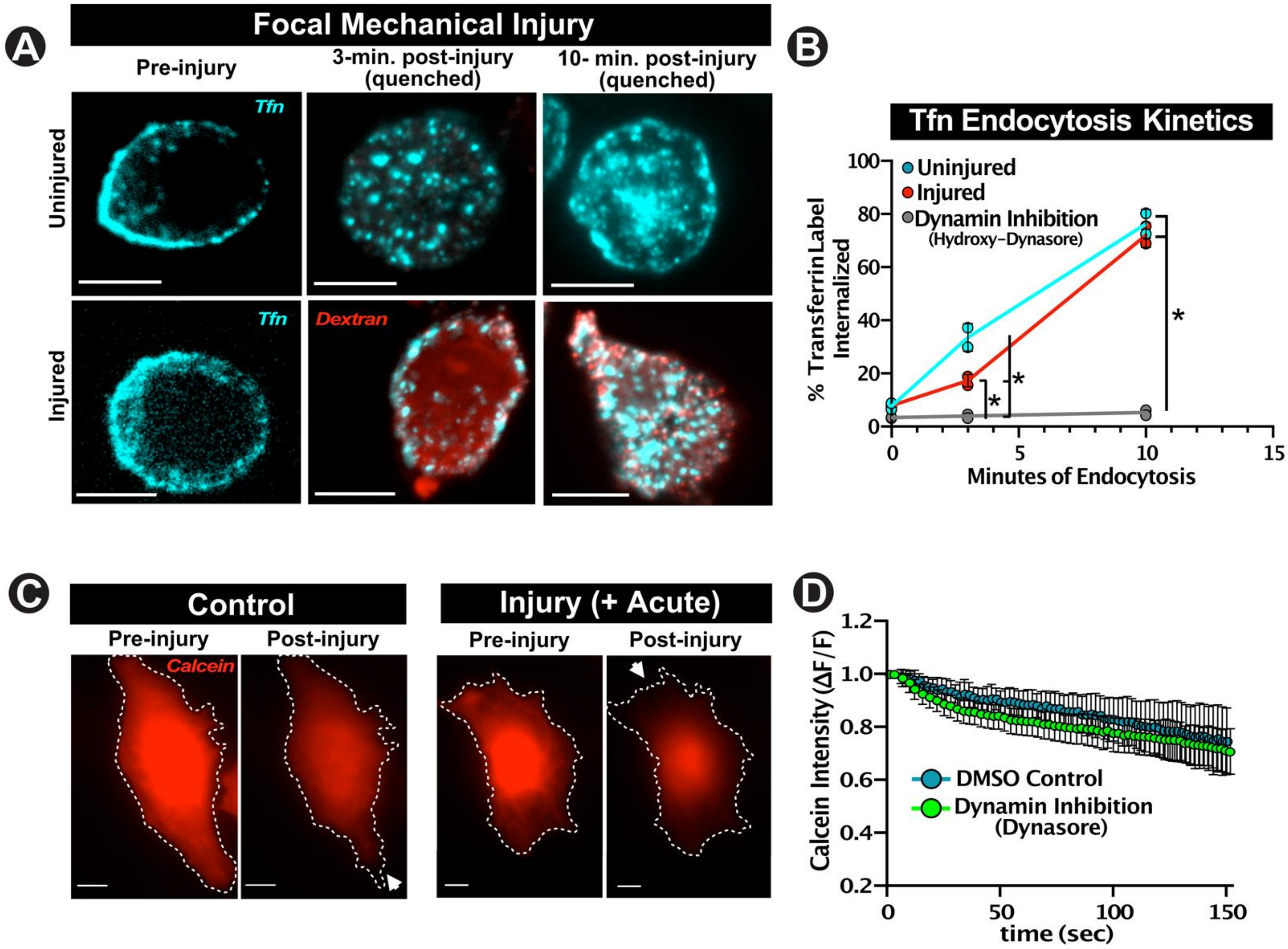

<u>Figure S3.</u> **PM endocytosis is transiently attenuated following mechanical PM injury**.

A. Images showing Transferrin (Tfn^{AF488}) PM-labelled cells immediately post-labelling (left panel), and at 3-minutes and 10-minutes post mechanical injury with BPB quenching of non-internalized PM fluorescence (bottom middle and right panels respectively, top panels are uninjured cells at those same timepoints). Bottom panel cells were scrape wounded in the presence of TRITC dextran, denoting cells that have been mechanically injured, enabling the monitoring of bulk endocytosis. **B.** Plot showing the kinetics of bulk endocytosis in uninjured cells (blue trace), dynamin-inhibited cells (hydroxy-dynasore, gray trace), and in mechanically injured cells (red trace) (*P<0.05 vs. specified condition) (3min. - *Control vs. Injured: p=0.014, Control vs. Dynamin Inhibition: p=.007) (10-min. - *Control vs. Dynamin Inhibition: p<0.001, Injured vs. Dynamin Inhibition: p<0.001). **C.** Images of Calcein-loaded cells pre/post focal mechanical injury in cells that are dynamin-inhibited (1st generation Dynasore) for <30 minutes (+ Acute), or treated with equivalent concentration of DMSO (control). **D.** Plot showing kinetics of Calcein loss following focal injury of cells treated as stated. Data (A,B) represents 2 separate experiments (n= 25-30 cells per repeat, total of 55-60 cells per condition), and for (C,D) 3 separate experimental repeats (15-20 cells per repeat per condition). Data indicate mean ± SEM. Kinetics analyses in D performed via 1-way repeated-measures ANOVA with post-hoc t-tests and Bonferroni correction. Differences across multiple treatments in in B were assessed via 1-way ANOVA. Scale bars-10 µm. (C) White arrows= site of focal mechanical injury.

Figure S4.

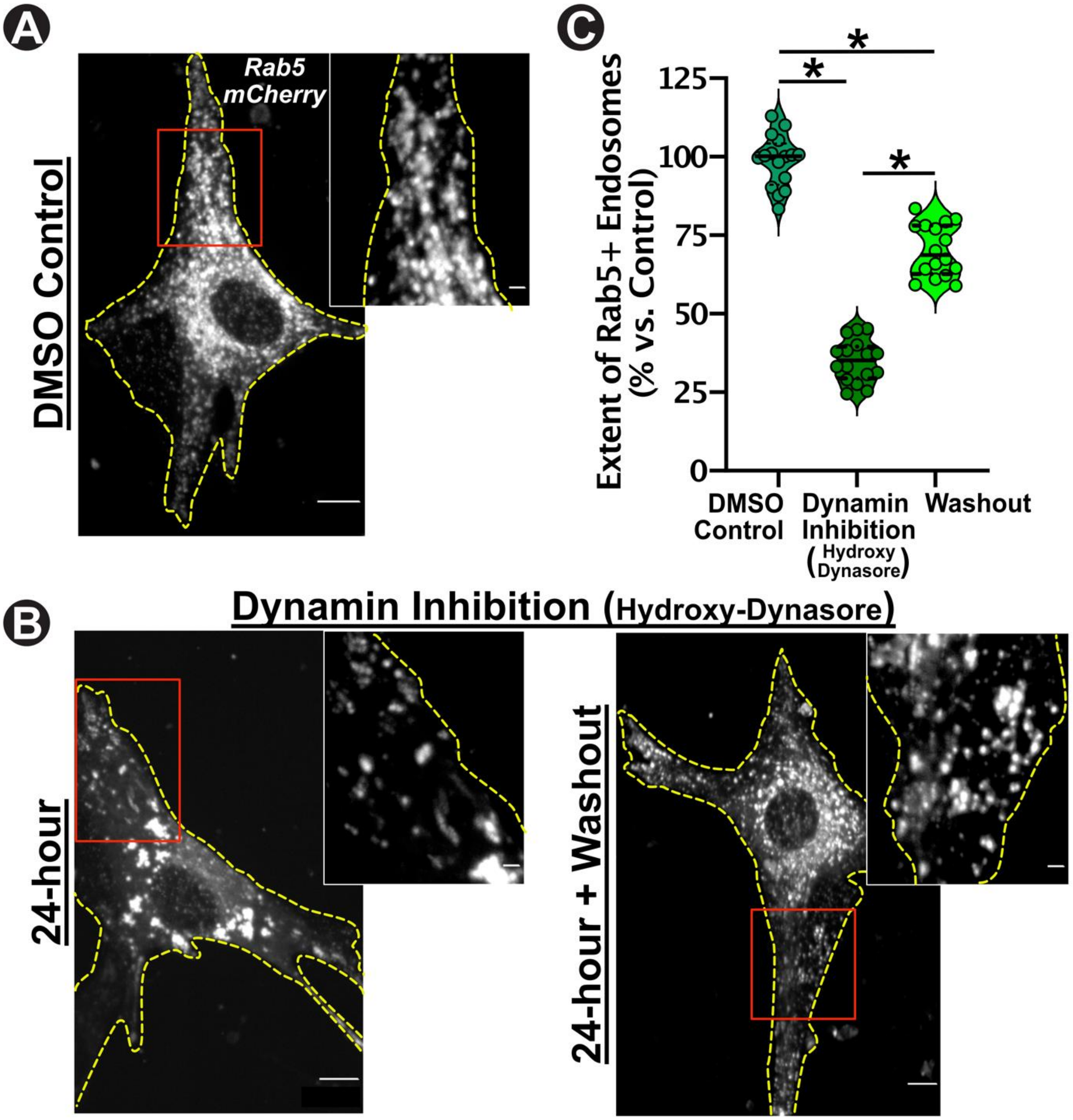

<u>Figure S4.</u> **Chronic inhibition of PM endocytosis depletes early endosome abundance**.
A,B. Confocal images of Rab5-mCherry-transfected cells (pseudocolored white). Inset images represent enlarged/zoomed regions at peripheral regions of the cell at the PM/coverslip interface to highlight Rab5+ early endosome abundance across conditions (DMSO-control - A, Hydroxy-Dynasore-treated cells, and Hydroxy-Dynasore-treated cells + washout - B). B. Cells were treated for 24 hours. Washout cells underwent 2 hour washout of Hydroxy-Dynasore prior to imaging. **C.** Quantification of proportion of early endosome abundance (relative to DMSO-control treated cells) across conditions (n = 16 cells per condition) (*p=.003 Dynamin Inhibition < DMSO control, p=.016 Dynamin Inhibition < Washout, p=.037 washout < DMSO control). Differences in early endosome abundance determined via 1-way ANOVA for multiple comparisons. Scale bars (A,B = 10 µm, insets = 2 µm). Data indicate mean $\pm$ SEM.
